# Synaptotagmin oligomers are necessary and can be sufficient to form a Ca^2+^‐sensitive fusion clamp

**DOI:** 10.1002/1873-3468.13317

**Published:** 2019-01-18

**Authors:** Sathish Ramakrishnan, Manindra Bera, Jeff Coleman, Shyam S. Krishnakumar, Frederic Pincet, James E. Rothman

**Affiliations:** ^1^ Department of Cell Biology Yale University School of Medicine New Haven CT USA; ^2^ Department of Clinical and Experimental Epilepsy UCL Queen Square Institute of Neurology London UK; ^3^ Laboratoire de Physique Statistique Ecole Normale Supérieure Sorbonne Universités UPMC Univ Paris 06, CNRS PSL Research University Université Paris Diderot Sorbonne Paris Cité France

**Keywords:** calcium, fusion clamp, single‐vesicle analysis, SNARE proteins, Synaptotagmin

## Abstract

The buttressed‐ring hypothesis, supported by recent cryo‐electron tomography analysis of docked synaptic‐like vesicles in neuroendocrine cells, postulates that prefusion SNAREpins are stabilized and organized by Synaptotagmin (Syt) ring‐like oligomers. Here, we use a reconstituted single‐vesicle fusion analysis to test the prediction that destabilizing the Syt1 oligomers destabilizes the clamp and results in spontaneous fusion in the absence of Ca^2+^. Vesicles in which Syt oligomerization is compromised by a ring‐destabilizing mutation dock and diffuse freely on the bilayer until they fuse spontaneously, similar to vesicles containing only v‐SNAREs. In contrast, vesicles containing wild‐type Syt are immobile as soon as they attach to the bilayer and remain frozen in place, up to at least 1 h until fusion is triggered by Ca^2+^.

## Abbreviations

**DOPS**, 1,2‐dioleoyl‐sn‐glycero‐3‐ (phospho‐l‐serine)

**NBD‐DOPE**, 1,2‐dipalmitoyl‐sn‐glycero‐3‐phosphoethanolamine‐N‐(7‐nitro‐2‐1,3‐benzoxadiazol‐4‐yl)

**OG**, Octylglucoside

**PSM**, pore‐spanning bilayer membrane

**SUV**, small unilamellar vesicle

**Syt**, Synaptotagmin

**TCEP**, Tris(2‐carboxyethyl)phosphine hydrochloride

**vSUV**, VAMP2‐ containing SUV

In the accompanying paper 1, we report that exactly six SNAREpins, symmetrically positioned in a circular array, underlie each synaptic vesicle that is docked to the plasma membrane as it remains clamped and awaiting a Ca^2+^ ion signal to trigger its fusion. We further find that the precise positioning of these half‐zippered SNARE complexes is determined by an underlying ring of the calcium‐sensor protein Synaptotagmin1 (Syt1), analogous to the *in vitro* ring‐like oligomers observed with purified Syt1 protein in the presence of PIP_2_ or analogous compounds 2, 3, 4. This follows from the finding that the symmetrical organization of the fusion machinery under synaptic vesicles is lost or prevented by an engineered point mutation (F349A) in the polymerizing Syt1 C2B domain that destabilizes the oligomers without affecting any other known molecular properties 5.

As the Syt1 rings assembled on phospholipid surfaces are disassembled by Ca^2+^ 2, 4, we hypothesized that the assembly of a Syt ring provides the core mechanism of this fusion clamp, and its disassembly by Ca^2+^ enables synchronous vesicle release 2, 4, 6. Indeed, expressing the ring‐destabilizing F349A mutant in neuroendocrine (PC12) cells dominantly and dramatically increases spontaneous release 5, suggesting that Syt1 oligomers play a necessary role in providing a reversible fusion clamp.

In this paper we provide complementary evidence, using a biochemically‐defined system involving only SNAREs and Syt1. In doing so, we utilize a novel suspended bilayer design 7 in which bilayers span an array of micro‐fabricated pores (5 μm diameter) with aqueous exposure on both sides, enabling them to be observed in a parallel fashion 7, 8. We employ small unilamellar vesicles (SUVs) to mimic synaptic vesicles. These are added from one side of the suspended bilayers (which serve to mimic the plasma membrane). We then monitor the fate of the docked vesicles in real‐time, yielding statistically meaningful results with automated analysis 7, 8.

We find that Syt1 alone provides a stable fusion clamp that is efficiently reversed by Ca^2+^, and that this clamp is prevented by a ring‐destabilizing mutation. This suggests that ring‐like oligomers of Syt1 are both necessary and in some cases may even be sufficient for clamping and release.

## Materials and methods

### Materials

The following cDNA constructs, which have been previously described 9, 10, 11, were used in this study: full‐length VAMP2 (VAMP2‐His^6^, residues: 1–116); full‐length t‐SNARE complex (mouse His^6^‐SNAP25B, residues: 1–206 and rat Syntaxin1A, residues: 1–288), and Syt (rat Syt1‐His^6^, residues: 57–421). The F349A mutant was created in the Syt1 background using the QuickChange mutagenesis kit (Agilent Technologies, Santa Clara, CA, USA). Lipids, 1,2‐dioleoyl‐snglycero‐3‐phosphocholine, 1,2‐dioleoyl‐sn‐glycero‐3‐ (phospho‐l‐serine) (DOPS), 1,2‐dipalmitoyl‐sn‐glycero‐3‐phosphoethanolamine‐*N*‐(7‐nitro‐2‐1,3‐benzoxadiazol‐4‐yl) (NBD‐DOPE), phosphatidylinositol 4, 5‐bisphosphate (PIP2) were purchased from Avanti Polar Lipids (Alabaster, AL, USA). ATTO647N‐DOPE was purchased from ATTO‐TEC, GmbH (Siegen, Germany).

### Protein purification

The SNARE and Syt1 proteins were expressed and purified as described previously 9, 10, 11. Briefly, the proteins were expressed in *Escherichia coli* strain Rosetta2(DE3) (Novagen, Darmstadt, Germany) and cells were lysed with a cell disruptor (Avestin, Ottawa, CA, USA) in HEPES buffer containing 25 mm HEPES, pH 7.4, 400 mm KCl, 4% TritionX‐100, 10% glycerol, 0.5 mm Tris(2‐carboxyethyl)phosphine hydrochloride (TCEP) and 1 mm phenylmethylsulfonyl fluoride. Samples were clarified using a 45Ti rotor (Beckman Coulter, Brea, CA, USA) at 140 000 ***g*** for 30 min and incubated with Ni‐NTA agarose (Qiagen, Valencia, CA, USA) for 4–16 h at 4 °C. The resin was subsequently washed in the same buffer, except that Triton X‐100 was replaced with 1% Octylglucoside (OG) and 50 mm Imidazole was also added. For Syt1 protein, the resin was resuspended in HEPES buffer containing 1% OG supplemented with 10 μg·mL^−1^ DNaseI, 10 μg·mL^−1^ RNaseA, and 10 μL of benzonase (2000 units) and incubated at room temperature for 1 h, followed by a quick rinse with 10 mL of high salt buffer (25 mm HEPES, 1 m KCl, 0.5 mm TCEP) to remove the nucleotide contamination. After washing (three column volumes) the bound protein was eluted in the same HEPES buffer containing 1% OG and 300 mm Imidazole. The Syt1 protein was additionally cleaned using Mono‐S cation exchange chromatography. The protein concentration was determined using a Bradford Assay (BioRad, Hercules, CA, USA) with BSA as a standard and protein purity was verified using SDS/PAGE analysis with Coomaisse stain.

### Liposome reconstitution

Proteoliposomes containing t‐SNAREs (preformed Syntaxin/SNAP25 complexes) or VAMP2 (± Syt1) were prepared using rapid detergent (1% OG) dilution and dialysis, followed by a discontinuous Nycodenz gradient as previously described 9, 10. The lipid composition was 80 (mole)% POPC, 15% DOPS, 3% PIP2 and 2% NBD‐PE for t‐SNARE liposome and 88% POPC, 10% PS and 2% ATTO647‐PE for Syt1/VAMP2 liposomes. To achieve the desired final protein density in the proteoliposomes, we used an input protein: lipid ratio of 1 : 400 for t‐SNARE, 1 : 500 for VAMP2 and 1 : 250 for Syt1. This was based on well‐established parameters 12, namely that the reconstitution efficiency for SNAREs and Syt1 is 50–60% (densitometry analysis of the proteoliposomes) and only 50–55% of the proteins are externally oriented (chymotrypsin protection analysis). Based on the densitometry analysis of Coomaisse‐stained SDS gels (Fig. S1), we estimated that the outward‐facing VAMP2 and Syt1 protein densities were 13 ± 2 and 28 ± 4 copies per vesicle, respectively (Fig. S1).

### Single‐vesicle fusion analysis

Single‐vesicle fusion measurements were performed with suspended lipid bilayers as described previously 7, 8. Briefly, a pore‐spanning lipid bilayer was formed by bursting t‐SNARE‐containing giant unilamellar vesicles (prepared using the osmotic shock protocol described recently 13) onto plasma cleaned Si/SiO_2_ chips containing 5 μm diameter holes spaced 5 μm apart in presence of 5 mm MgCl_2_. Before each vesicle fusion assay, the homogeneity and fluidity of the t‐SNARE containing bilayers was confirmed using fluorescence recovery after photo‐bleaching. Consistent with a fluid bilayer, the average diffusion coefficient of the lipid was calculated to be 2.2 ± 0.8 μm^2^·s^−1^ (Fig. S1). In some experiments, we also instead labeled the t‐SNAREs with Alexa‐488 and confirmed protein mobility as described previously 7.

**Figure 1:**
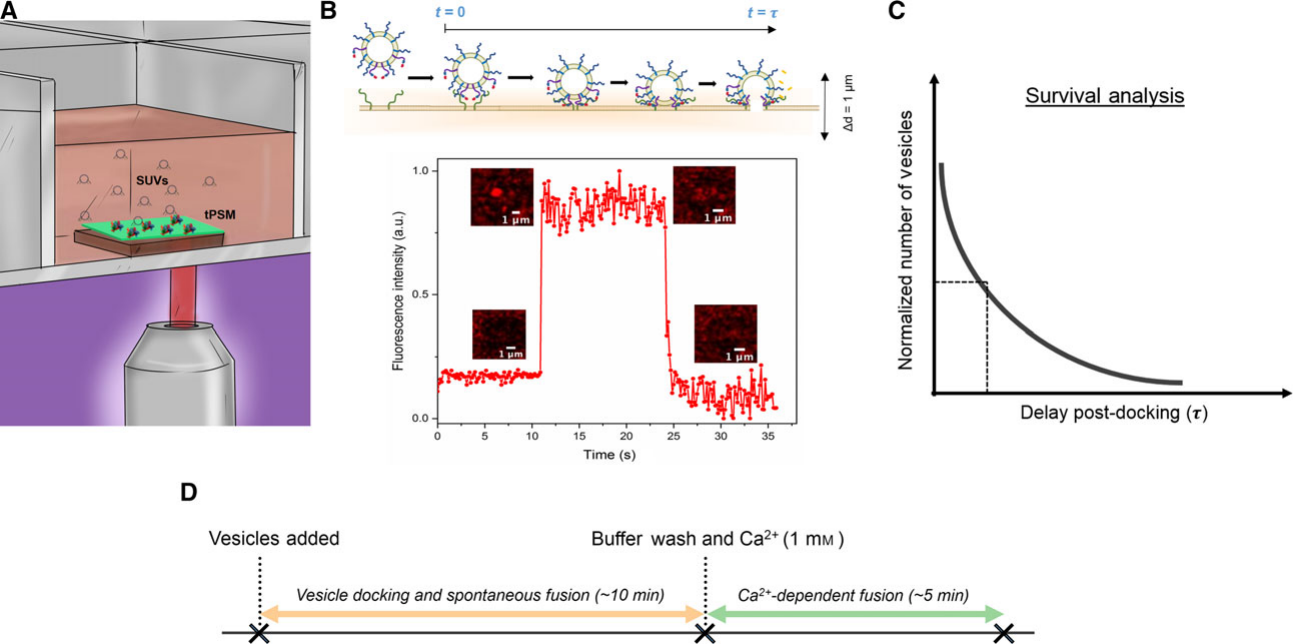
Overview of the single‐vesicle docking and fusion analysis with a pore‐spanning bilayer (A) VAMP2 (± Syt1)‐containing SUVs are added to the t‐PSM from the top and monitored, using a confocal microscope from the bottom. (B) The docking, mobility and the fusion of the SUVs with the t‐PSM is tracked, using the fluorescence marker (ATTO647N‐PE) included in the SUVs. A representative fluorescence trace showing a behavior of a typical vesicle is shown. The time between docking and fusion (τ) is measured for each docked vesicle and the results for the whole population are presented as a survival curve (C). (D) To monitor the fate of substantial numbers of individual vesicles from each experimental trial, the SUVs were allowed to interact with t‐PSM for 10 min and critical parameters, including vesicle docking, mobility of the docked vesicles and Ca^2+^‐independent spontaneous fusion were acquired. The chamber was subsequently washed with buffer and then Ca^2+^ (1 mm) was added from the top to record (~ 5 min) Ca^2+^‐dependent fusion events.

Typically, ~ 100 nm (final lipid concentration) v‐SUVs diluted in running buffer (25 mm HEPES, 140 mm KCl, 1 mm DTT) were introduced into the chamber and allowed to interact with the t‐SNARE bilayer for ~ 10 min. A laser scanning confocal microscopy equipped with 647‐nm diode laser was used to track the docking, diffusion and fusion of individual vesicles (Fig. 1). All experiments were performed at 37 °C and images were acquired at 147 milliseconds between frames. The images were then analyzed using a custom‐made fusion analyzer Software 7 to automatically detect and estimate the diffusional mobility of individual docked vesicles, to count the number of docked vesicles that remained attached but unfused and the number that had undergone fusion. The results are presented as a ‘survival curve’ which plots the fraction of vesicles that docked on to the planar bilayer and remained unfused as a function of the time elapsed after docking (Fig. 1). Fusion was attested by a burst and then a rapid decrease in fluorescence intensity as the fluorescent PE from the vesicle diffuses away. After the initial 10 min interaction phase, the chamber was washed (three‐times) with running buffer to remove free vesicles and then CaCl_2_ (1 mm final concentration) was added to enable Ca^2+^‐triggered fusion of the remaining docked vesicles. The number of fused (and the remaining un‐fused) vesicles was estimated after ~ 5 min. Note: We observed some t‐SNAREs aggregation at the edge of the holes indicating interaction with the neighboring substrate. Similarly, some vesicles bind at the edge of the holes and remain immobile. These are probably not representative of vesicles bound to the free‐floating membrane. Hence, we excluded these vesicles and picked only the centrally docked ones for analysis.

### Single‐vesicle docking analysis

For the docking analysis, ~ 100 nm (total lipid concentration) of SUVs diluted in running buffer (25 mm HEPES, 140 mm KCl, 1 mm DTT) were introduced into the chamber and allowed to interact with the t‐SNARE bilayer. After a 10‐min incubation, the bilayer was thoroughly washed with running buffer (3× minimum) and the number of docked vesicles were counted. For an unbiased particle count, we employed a custom‐written algorithm to count particles from top‐left to bottom‐right that ensures every spot is counted only once 7, 8. To get an accurate count of the docked vesicles, we used VAMP2 protein with mutations in the C‐terminal half (L70D, A74R, A81D and L84D; termed VAMP2‐4X) that eliminates fusion without impeding the docking process 14, 15.

## Results

The outline of the experimental approach is shown in Fig. 1. SUVs are prepared with fluorescently labeled lipid (2% ATTO647‐PE) and typically contain the v‐SNARE VAMP2 with or without Syt1 (Fig. S1). The SUVs are added from the top of the chamber and observed from below (Fig. 1B). When a vesicle docks, it appears as a discrete puncta (taken as time zero for each such vesicle). When it fuses, the puncta is replaced by locally dispersed fluorescence that rapidly diffuses away (Fig. 1C). The vesicle may or may not be mobile on the surface between docking and fusion. We monitor large ensembles of vesicles (~ 500 vesicles per condition) to determine the percent remaining unfused (‘survival analysis’) as a function of time elapsed after docking and present the results as a survival curve (Fig. 1D) which provides a measure of the kinetics of fusion following docking.

To simplify the experimental approach and bypass the requirement of SNARE‐assembling chaperones (Munc18 and Munc13 16, 17, 18), we employed preformed t‐SNAREs (1 : 1 complexes of Syntaxin1 and SNAP‐25) in the planar bilayers (Fig. S1). Triton extracts from native synaptic vesicles, contain synaptophysin hexamer in a stable complex with 12 copies of VAMP2 19, which presumably enter the fusion process together. To mimic this (in the absence of Synaptophysin), we chose reconstitution conditions resulting in an average of ~ 12 copies of outward‐facing VAMP2 per SUV. We reconstituted ~ 28 copies of outward‐facing Syt1 in the SUVs, a value chosen for two reasons. First, synaptic vesicles contain 15–22 copies of Syt1 20, 21 and second, *in vitro* Syt1 rings typically contain 15–25 copies of Syt1 2.

**Figure 2:**
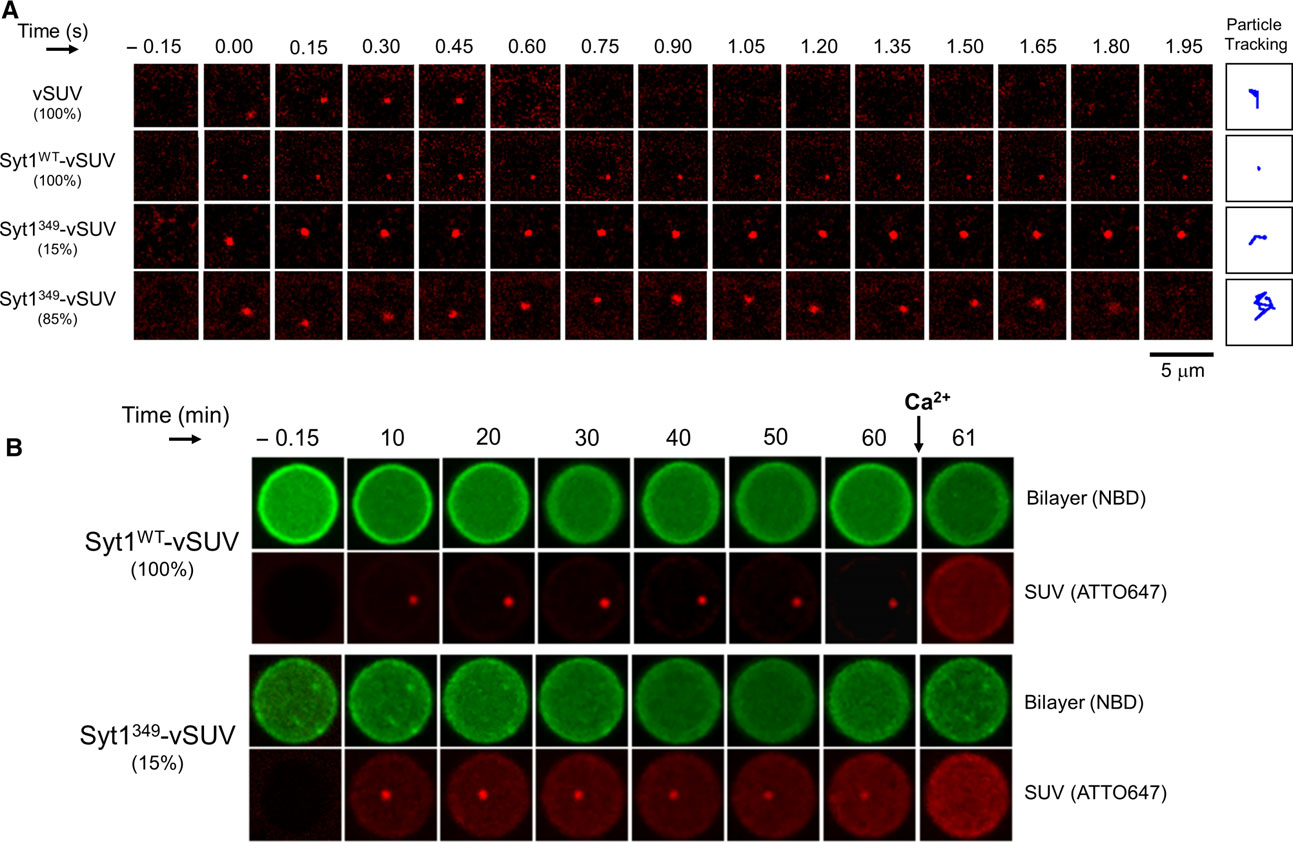
The mobility of docked vesicles containing VAMP2 with or without Syt. (A) Representative time‐lapse fluorescence (ATTO647N) images of a single docked SUVs show that all vSUVs are diffusively mobile upon docking and fuse spontaneous with a half‐time of ~ 1 s (Fig. 3). In contrast, the vSUVs with Syt1^WT^ (Syt1^WT^‐vSUV) are stably docked in‐place and largely immobile and do not fuse, until triggered by Ca^2+^. When Syt1 oligomerization is compromised with a targeted mutation (F349A), a large majority (85%) of these SUVs (Syt1^349^‐vSUV) are mobile and spontaneous fuse similar to vSUVs, while a minority (15%) are immobile upon docking and never fuse. Representative video files corresponding to these images are included as Videos [Link], [Link], [Link], [Link]. (B) The immobile fractions of Syt1^WT^‐vSUV (100%) and Syt1^349^‐vSUV (~ 15%) remain stably clamped and Ca^2+^‐sensitive for at least 1 h, the observation period limited by photo‐bleaching and bilayer stability. In such experiments, designed to test the stability of docked vesicles, it was necessary to minimize fluorescence bleaching by only imaging every 10 min prior to Ca^2+^‐addition, and every 1 min post‐Ca^2+^‐addition. The NBD‐fluorescence included on the pore‐spanning bilayer was recorded at each time point to verify the continued integrity of the pore‐spanning suspended bilayer.

**Figure 3:**
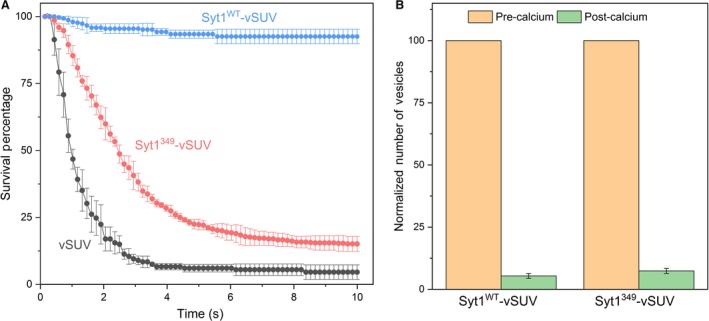
Syt1 oligomers form a Ca^2+^‐sensitive fusion clamp. (A) The cumulative docking‐to‐fusion delays represented as the survival percentage shows that vSUVs spontaneous fuse with a half‐time of ~ 1 s, but Syt1^WT^‐vSUV remain stably docked. Destabilizing the Syt1 oligomers (Syt1^349^) destabilizes the fusion ‘clamp’ with the majority of vesicles proceeding to fuse spontaneously. (B) Clamped SUVs containing Syt1^WT^ or Syt1^349^ are triggered to fuse by Ca^2+^ (1 mm). End‐point analysis at 5 min post‐Ca^2+^‐addition shows that > 90% of all clamped vesicles fuse following Ca^2+^ addition. Representative video file corresponding to fluorescence change associated with Ca^2+^‐triggered exocytosis is shown in Videos [Link], [Link]. The average values and standard deviations at each time point from three independent experiments are shown for each condition. In total, > 500 vesicles were analyzed for each condition.

When SUVs contained only VAMP2, > 95% of these vesicles (termed ‘vSUV’) that had docked to a t‐SNARE containing pore‐spanning bilayer membrane (t‐PSM) rapidly and spontaneously fused after a period of diffusion on the PSM surface (Fig. 2A). The half‐time for survival was ~ 1 s (Fig. 3A). Virtually every docked v‐SUV was diffusively mobile (Fig. 2A). Each such vSUV continued to diffuse until, within one video frame (each frame was about 150 ms), diffusion ceased and fusion occurred. Most likely, fusion results immediately when a handful of SNAREpins are formed.

Adding Syt1 wild‐type (Syt1^WT^) to these vesicles resulted in a completely different behavior. Inclusion of Syt1 increased the number of docked vesicles (Table 1) but now, these vesicles (termed ‘Syt1^WT^‐vSUV’) were immobile following docking to the bilayer surface (Figs 2A and 3A).The vesicles rarely fused over the initial 10 min observation period until Ca^2+^ (1 mm) was added after which the vast majority fused within the 5‐min time allowed (Fig. 3B). This behavior is referred to as ‘clamped’. In fact, these vesicles remain stably clamped and Ca^2+^‐sensitive for hours (Figs 3B and S2). Note: we monitored these docked vesicles up to 3 h (currently limited by the stability of the suspended bilayer and photo‐bleaching; Fig. S2). In the present studies, Ca^2+^ was simply added from the top (Fig. 1) and required 5–10 s to diffuse to the bilayer, so information on the precise kinetics of Ca^2+^‐triggered fusion is presently lacking.

**Table 1:** Quantification of docking of vSUVs (± Syt1) in the presence or absence of PIP2 in the suspended bilayer. Inclusion of Syt1 (both WT and F349A) increased the docking of vSUVs to the t‐PSM. The vesicle attachment is likely mediated by the interaction of the polybasic motif of the C2B domain with the negatively charged lipids, namely PIP2 (3%) and DOPS (15%) 22, 23, 24. In support of this premise, exclusion of PIP2 significantly lowered the number of docked vesicles. In all cases, a mutant form of VAMP2 (VAMP2‐4X) which eliminated fusion was used to unambiguously estimate the number of docked vesicles after the 10 min interaction phase. The average and standard deviation from three independent experiments are shown

Vesicle type	Number of docked vesicles per 100 μm^2^	
	+ PIP2	− PIP2
vSUV	1.4 ± 0.2	N.D.
Syt1^WT^‐vSUV	16 ± 2.0	8.0 ± 2.0
Syt1^349^‐vSUV	28 ± 5.0	12 ± 3.0

Syt1 is known to attach to the bilayer by binding PIP_2_ 22, 23, 24. As expected, when PIP2 was omitted from the tPSM bilayers, the number of docked vesicles was greatly reduced compared to bilayers containing PIP_2_ (Table 1). Notably, the remaining docked Syt1^WT^‐SUVs were no longer clamped (Fig. S3), but rather fused spontaneously, similar to vSUVs. This shows that clamping in this reduced system requires PIP_2_, which is in fact physiologically required for assembly of the readily releasable pool 25, 26. PIP_2_ binds to the polybasic surface of Syt1, capturing the synaptic vesicle 22, 24. This same interaction also triggers Syt1 to polymerize into ring‐like oligomers *in vitro* 2, 4, which we have hypothesized is the central structure responsible clamping *in vivo*.

To examine if the ‘clamping’ behavior observed in the present simplified system is linked to the formation of Syt1 oligomers, we replaced the wild‐type Syt1 with a well‐characterized ring‐destabilizing point mutant, F349A (referred to as ‘Syt1^349^‐vSUVs’). Now, the vast majority of the vesicles (~ 85%) diffuse freely and fused spontaneously, in the absence of Ca^2+^ (Fig. 2A), suggesting that oligomerization of Syt1 is indeed required for clamping in this system. A minority (~ 15%) of the docked Syt1^349^‐vSUVs were indistinguishable in their behavior from Syt1^WT^ containing vesicles. These vesicles were immobile and remained unfused in Mg^2+^, but then efficiently fuse upon Ca^2+^ addition (Figs 2 and 3).

The ability of Syt1 to bind PIP_2_ is unaffected by the F349A mutation 5 and correspondingly, we observed robust docking of the Syt1^349^‐vSUVs (Table 1). Interestingly, these vesicles diffused freely until they fused (Fig. 2A), similar to the vSUVs. This is in stark contrast to the lack of diffusion of the clamped Syt1^WT^‐vSUVs (Fig. 2), implying that the assembly of Syt1 oligomers is the main factor causing immobilization (as it also seems to be for clamping). This immobilization is to be expected, since each subunit of a 15–25 member Syt1 oligomer 2 should be able to simultaneously attach to the PSM *via* PIP_2_.

Finally, as a control, vesicles containing only Syt1^WT^ and no VAMP2 were found to dock, but never fuse, even after Ca^2+^ was added (Fig. S4). They remained immobile for the entire observation period (Fig. S4). This finding rules out the possible caveat that Ca^2+^‐triggered fusion results not from SNAREpins, but instead an artifact of PS in the opposing lipid bilayers interacting directly with calcium ions. This possibility is also ruled out by the identical behavior (Fig. S4) of v‐SUVs containing the VAMP4X mutant 14, 15 which can form SNAREpins but is prevented from terminal zippering, and thus, fusion.

## Discussion

A principal finding is that Syt alone can stably and reversibly clamp vesicles in this reduced and fully‐defined system for at least 1 h. While physiological analyses have clearly identified a role for Syt1 as part of the fusion clamp 27, 28, 29, previous *in vitro* analysis in which membrane‐anchored Syt1 and SNAREs were mixed in the absence of Complexin have failed to establish a stable clamp of the kind that is readily demonstrated in the presence of Complexin 30, 31, 32, 33. The main difference between our study and all the others is that we limit the number of v‐SNAREs per vesicle (to ~ 12 copies) whereas the others seek to mimic the VAMP2 content of native synaptic vesicles (~ 70 copies). As one possibility, we suggest that Syt is sufficient to produce a stable clamp for a limited number of SNAREpins and that Complexin may be uniquely required (among its other roles) for the excess SNAREpins. We note that a previous study suggested that Syt alone can produce a stable clamp 34; however, this utilized the soluble cytoplasmic domain of Syt1 which in excess can effectively cover liposomal surfaces, rendering them non‐fusogenic.

The other principal finding of our study is that in our reduced system, the fusion clamp is largely ablated when Syt1 oligomerization is compromised by the F349A mutation. The majority of vesicles containing the mutant Syt1 (and v‐SNAREs) diffuse freely on the bilayer until they fuse spontaneously, similar to vesicles containing only the v‐SNAREs. This dramatically contrasts with the behavior of wild‐type Syt1, where the vesicles are immobile as soon as they attach to the bilayer and remain frozen in place until they bind calcium and then fuse, all without ever moving. The simplest explanation is that a ring‐like oligomer of Syt1^WT^ forms under the vesicle, immobilizing it *via* numerous contacts with the bilayer; but the mutant Syt1 fails to form these oligomers in most cases and the SNAREpins are therefore not stably clamped. As with vesicles lacking Syt1, the vesicles now diffuse until multiple SNAREpins have formed, pinning them down and fusing them. How Syt1 oligomers produce a stable clamp in our system is still unclear. One possibility is that Syt1 oligomers act as a ‘washer’ (or spacer) to sterically block fusion 2. Additionally but perhaps alternatively, Syt1 oligomers could bind and organize the SNAREs in a stable, partially assembled state as outlined in the buttressed ring model 6. Further experiments, including detailed mutational analysis, is required to dissect the precise molecular mechanism of the fusion clamp observed in this system.

It should be pointed out that the stable clamp by Syt1 could well be produced by oligomers (partial rings) as distinct from complete rings, and the dramatic loss of clamping with the mutant Syt1 could be due to its compromised ability to form oligomers of sufficient size, as distinct from completed rings. Further studies in which completed rings could be visualized by electron microscope methods, both in cell‐free systems and in cells *in situ,* will be needed to rigorously make this distinction. However, in light of the circularly symmetrical arrangement under synaptic‐like vesicles reported in neuroendocrine cells in the accompanying paper 1, it seems very likely that completely formed Syt1 rings are the basis of the stable clamp in the readily releasable pool of synaptic vesicles that enables synchronous synaptic transmission to keep pace with the action potential.

## Author contributions

SR, MB designed experiments, collected and analyzed/interpreted data. JC provided new reagents. SSK, FP and JER designed the experiments, provided supervision, analyzed/interpreted data and wrote the manuscript. All authors read and revised the manuscript.

## Supporting information


**Fig. S1.** (A) The proteoliposomes were prepared using a detergent dilution‐dialysis method, followed by a Nycodenz float‐up. The proteoliposomes were analyzed using SDS/PAGE analysis and visualized using Coomaisse stain. The protein density of the liposomes (with the loading amounts as control) was used to estimate the copy number of each protein per vesicle. (B) The fluorescence recovery after photo‐bleaching (FRAP) of the included NBD‐fluorophore was used to check the quality of the t‐SNARE containing the pore‐spanning suspended bilayer.
**Fig. S2.** Syt1^WT^ produced a stable fusion clamp.
**Fig. S3.** PIP2 is critical to both docking and the clamping of fusion by Syt1^WT^.
**Fig. S4.** Control experiments using Syt1^WT^ only or a nonfusogenic VAMP2 mutant (VAMP2‐4X) show that fusion under our experimental conditions strictly requires the SNARE proteins and a productive assembly of the SNARE complex.

**Video S1.** Video file corresponding to vSUV fusion shown in Figure 2A.

**Video S2.** Video file corresponding to Syt1^WT^‐vSUV clamp shown in Figure 2A.

**Video S3.** Video file corresponding to immobile/clamped fraction of Syt1^349^‐vSUV shown in Figure 2A.

**Video S4.** Video file corresponding to mobile/fusogenic fraction of Syt1^349^‐vSUV shown in Figure 2A.

**Video S5.** Video file corresponding to Ca^2+^‐associated fluorescence signal change of Syt1^WT^‐vSUV shown in Figure 3B.

**Video S6.** Video file corresponding to Ca^2+^‐associated fluorescence signal change of Syt1^349^‐vSUV shown in Figure 3B.
