# Supplementary material for: Synaptotagmin oligomers are necessary and can be sufficient to form a Ca2+‐sensitive fusion clamp
Source: FEBS Lett. 2019 Jan 18;593(2):154–62. doi: 10.1002/1873-3468.13317 (PMC6349546; doi:10.1002/1873-3468.13317)
Supplement: Supplementary file 1 — Fig. S1. (A) The proteoliposomes were prepared using a detergent dilution‐dialysis method, followed by a Nycodenz float‐up. The proteoliposomes were analyzed using SDS/PAGE analysis and visualized using Coomaisse stain. The protein density of the liposomes (with the loading amounts as control) was used to estimate the copy number of each protein per vesicle. (B) The fluorescence recovery after photo‐bleaching (FRAP) of the included NBD‐fluorophore was used to check the quality of the t‐SNARE containing the pore‐spanning suspended bilayer. Fig. S2. Syt1WT produced a stable fusion clamp. Fig. S3. PIP2 is critical to both docking and the clamping of fusion by Syt1WT. Fig. S4. Control experiments using Syt1WT only or a nonfusogenic VAMP2 mutant (VAMP2‐4X) show that fusion under our experimental conditions strictly requires the SNARE proteins and a productive assembly of the SNARE complex. [file FEB2-593-154-s001.docx]

Supplementary Information

**Synaptotagmin oligomers are necessary and can be sufficient to form a Ca^2+^-sensitive fusion clamp**

Sathish Ramakrishnan^1^, Manindra Bera^1^, Jeff Coleman^1^, Shyam S. Krishnakumar^1,2^, Frederic Pincet^1,3^ and James E. Rothman^1,2*^

^1^Department of Cell Biology, Yale University School of Medicine, New Haven, CT, 06520 USA. ^2^Department of Clinical and Experimental Epilepsy, UCL Queen Square Institute of Neurology, London, WC1 3BG, UK. ^3^Laboratoire de Physique Statistique, Ecole Normale Supérieure, PSL Research University; Université Paris Diderot Sorbonne Paris Cité; Sorbonne Universités UPMC Univ Paris 06, CNRS; 24 rue Lhomond, 75005 Paris, France.

*Correspondence: james.rothman@yale.edu

**
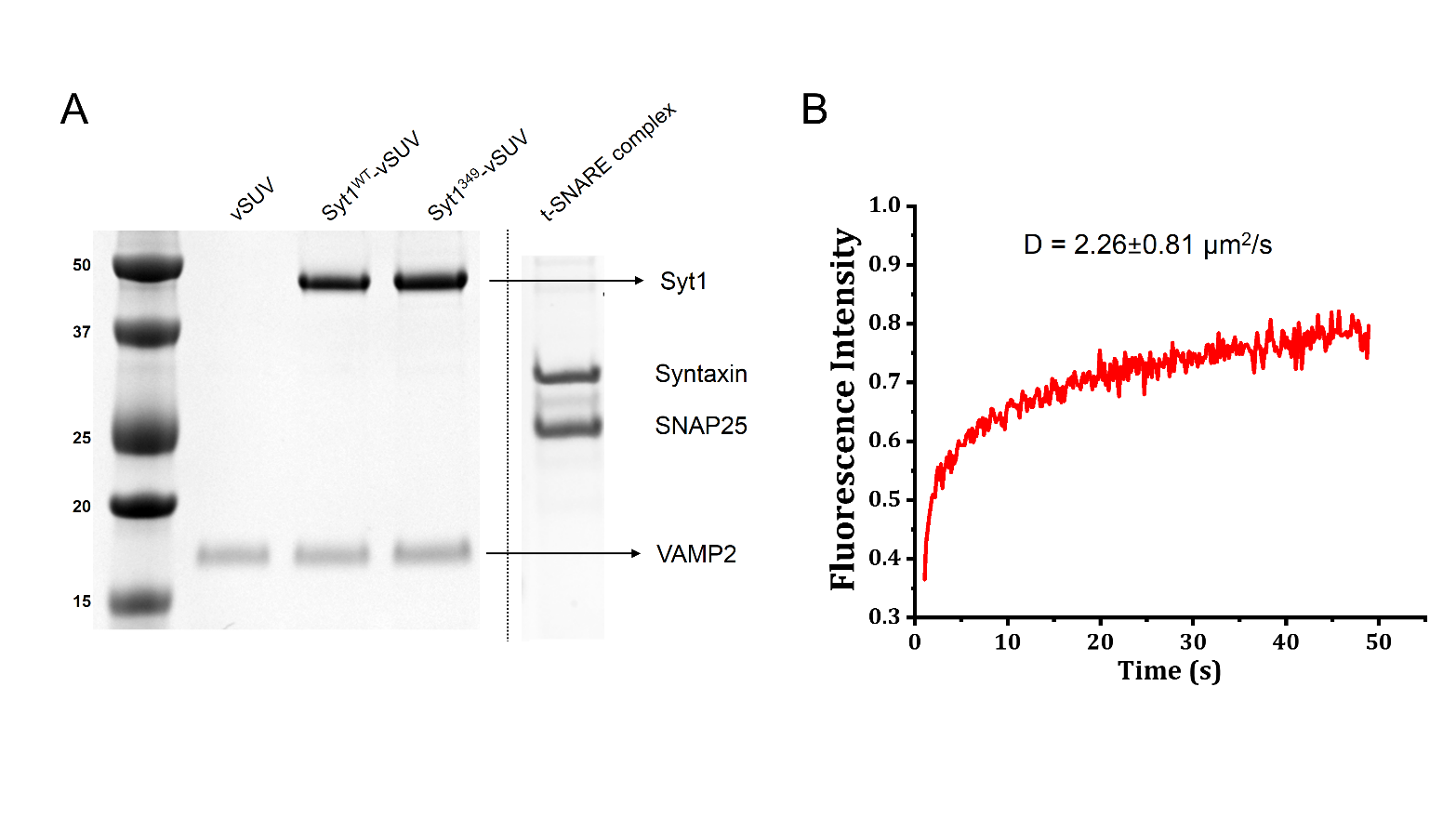
**

**Figure S1**. (A) The proteoliposomes were prepared using a detergent dilution-dialysis method, followed by a Nycodenz float-up. The proteoliposomes were analyzed using SDS-PAGE analysis and visualized using Coomaisse stain. The protein density of the liposomes (with the loading amounts as control) was used to estimate the copy number of each protein per vesicle. (B) The fluorescence recovery after photo-bleaching (FRAP) of the included NBD-fluorophore was used to check the quality of the t-SNARE containing pore-spanning suspended bilayer. Based on the fluorescence recovery curve, the average diffusion constant was calculated to be ~ 2.2 ± 0.8 µm^2^/s consistent with a relatively-fluid bilayer. Note: We applied this quality control to each and every bilayer formed before the addition of the SUVs.

**
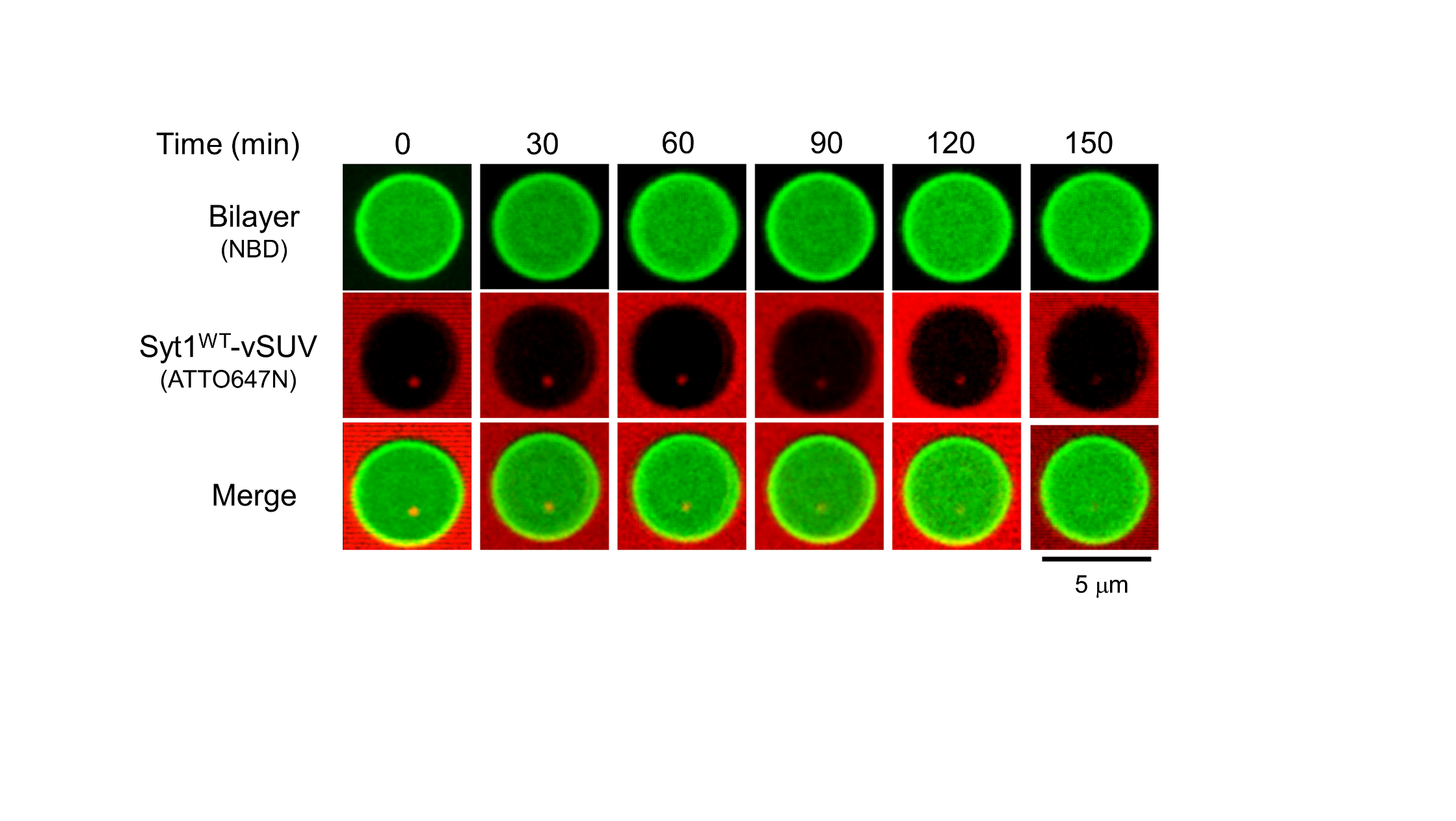
**

**Figure S2**. Syt1^WT^ produced a stable fusion clamp. Representative fluorescence (ATTO647N) image shows Syt1^WT^ containing vSUVs dock and stay immobile at the site of attachment for up to 3 hours. To obtain these long time-lapse image series, the vesicles were only imaged every 30 min for up to 150 min. At each time point, we also recorded the NBD-fluorescence to verify the stability of the bilayer. Images corresponding to a single 5 μm suspended bilayer is shown.

**
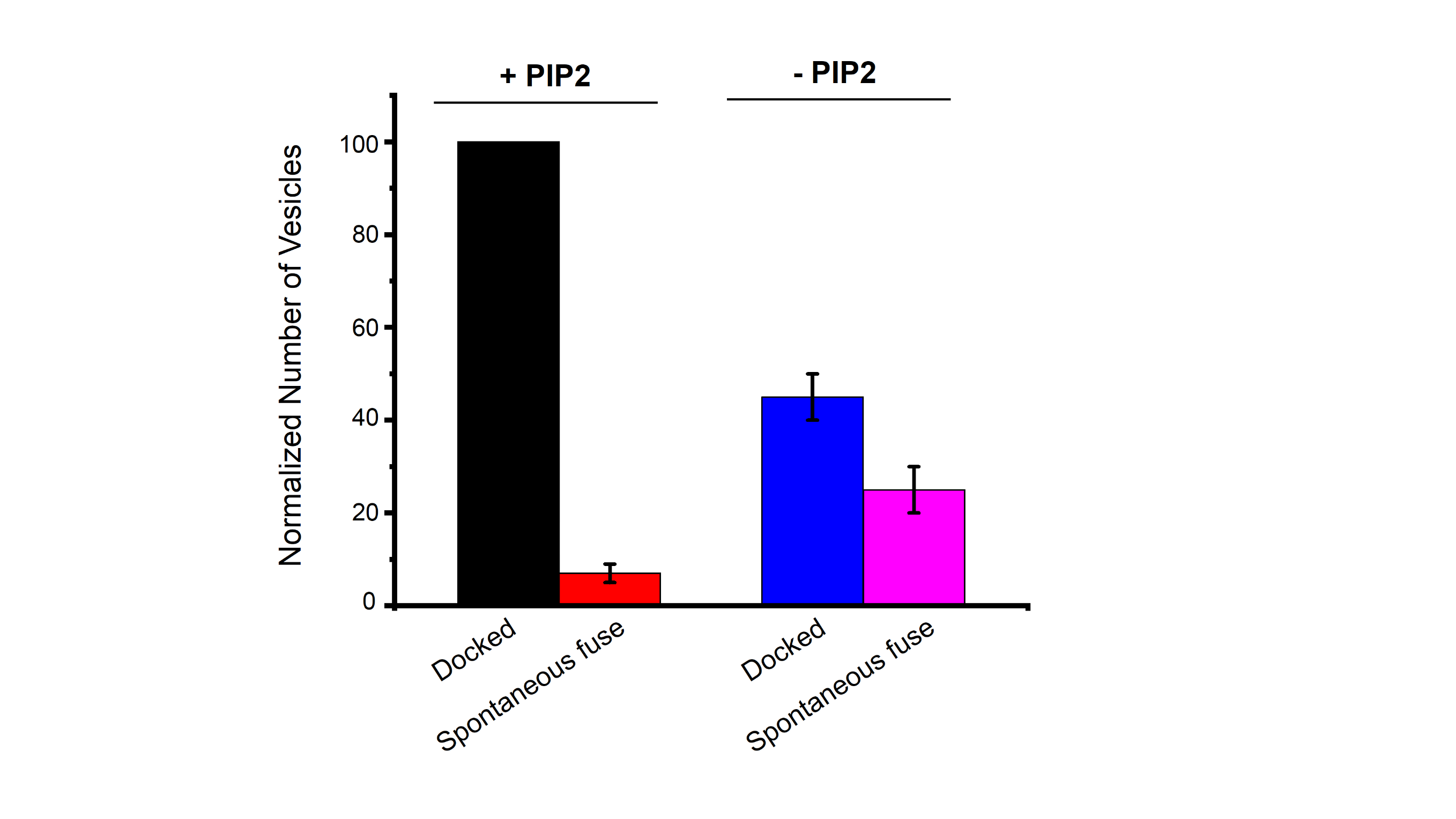
**

**Figure S3**. PIP2 is critical to both docking and the clamping of fusion by Syt1^WT^. Exclusion of PIP2 reduced the number of docked vesicles by ~50% and a majority of the docked vesicles were diffusively mobile on the bilayer and proceeded to fuse spontaneously. Average and standard deviations from 3 independent experiments, corresponding to ~250 vesicles are shown.

**
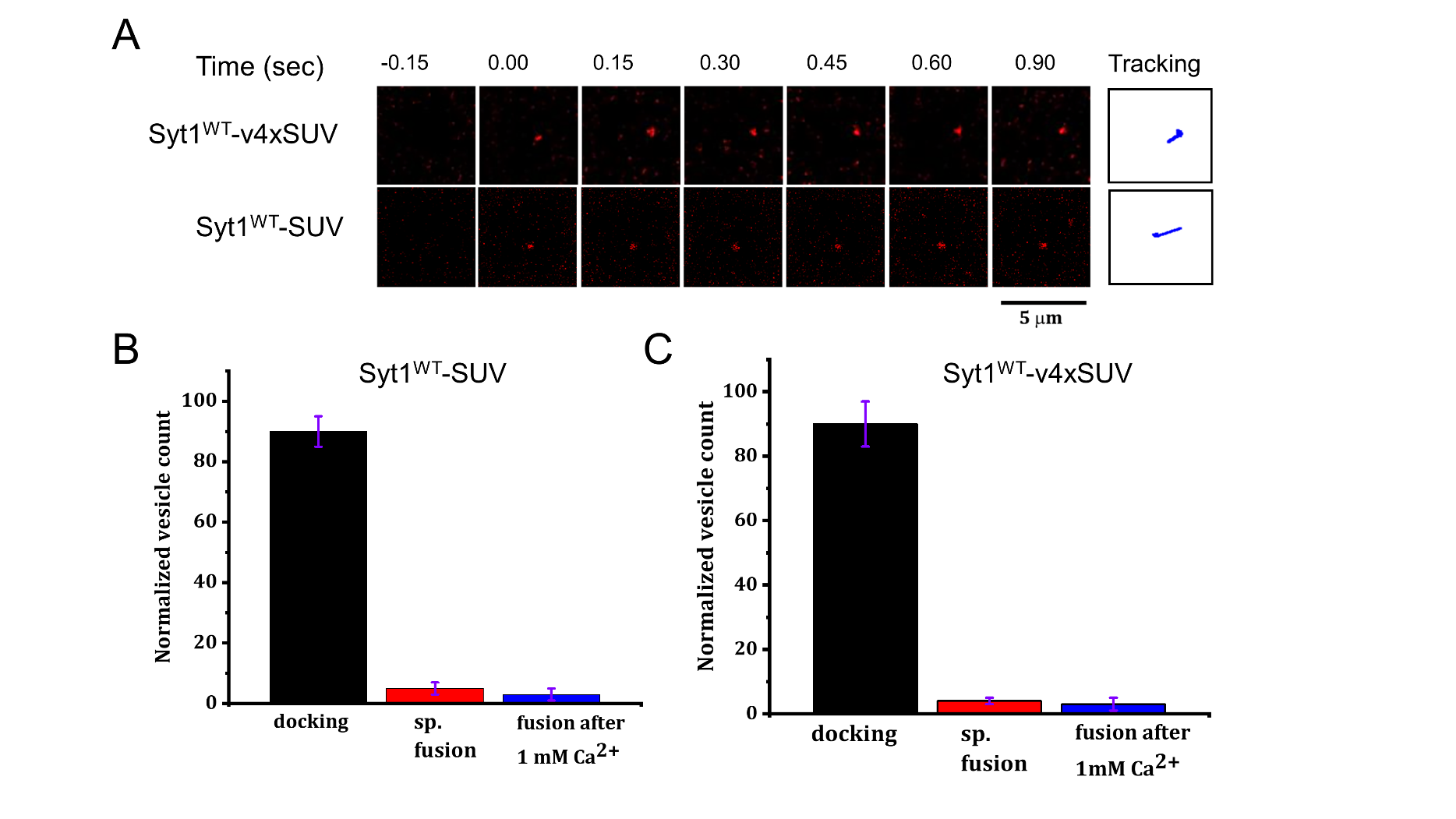
**

**Figure S4**. Control experiments using Syt1^WT^ only or a non-fusogenic VAMP2 mutant (VAMP2-4X) show that fusion under our experimental conditions strictly requires the SNARE proteins and a productive assembly of the SNARE complex. In both cases, the vesicles dock and remain largely immobile (A). There was very little (< 5%) to no spontaneous or Ca^2+^-evoked fusion of these docked vesicles (B, C). Data from three independent experiment, with a total of ~150 vesicles per condition is shown.
