# Supplementary material for: Synaptotagmin oligomers are necessary and can be sufficient to form a Ca2+‐sensitive fusion clamp
Source: FEBS Lett. 2019 Jan 18;593(2):154–62. doi: 10.1002/1873-3468.13317 (PMC6349546; doi:10.1002/1873-3468.13317)
Supplement: Supplementary file 8 [file FEB2-593-154-s008.docx]

Video S1. Video file corresponding to vSUV fusion shown in Figure 2A.

Video S2. Video file corresponding to Syt1^WT^-vSUV clamp shown in Figure 2A

Video S3. Video file corresponding to immobile/clamped fraction of Syt1^349^-vSUV shown in Figure 2A

Video S4. Video file corresponding to mobile/fusogenic fraction of Syt1^349^-vSUV shown in Figure 2A

Video S5. Video file corresponding to Ca^2+^-associated fluorescence signal change of Syt1^WT^-vSUV shown in Figure 3B.

Video S6. Video file corresponding to Ca^2+^-associated fluorescence signal change of Syt1^349^-vSUV shown in Figure 3B.
